# Association of MDM2 Overexpression in Ameloblastomas with *MDM2* Amplification and BRAF^V600E^ Expression

**DOI:** 10.3390/ijms25042238

**Published:** 2024-02-13

**Authors:** Konstantinos I. Tosios, Eleni-Marina Kalogirou, Ioannis G. Koutlas

**Affiliations:** 1 Department of Oral Pathology & Medicine and Hospital Dentistry, School of Dentistry, National and Kapodistrian University of Athens, 11527 Athens, Greece; 2 Faculty of Health and Rehabilitation Sciences, Metropolitan College, 15125 Athens, Greece; ekalogirou@mitropolitiko.edu.gr; 3 Division of Oral Pathology, School of Dentistry, University of Minnesota, Minneapolis, MN 55455, USA; koutl001@umn.edu

**Keywords:** odontogenic tumors, ameloblastoma, MDM2 protein, BRAF, p53, in situ hybridization, fluorescence

## Abstract

Ameloblastoma is a rare tumor but represents the most common odontogenic neoplasm. It is localized in the jaws and, although it is a benign, slow-growing tumor, it has an aggressive local behavior and high recurrence rate. Therefore, alternative treatment options or complementary to surgery have been evaluated, with the most promising one among them being a targeted therapy with the *v-Raf* murine sarcoma viral oncogene homologue B (*BRAF*), as in ameloblastoma the activating mutation V600E in BRAF is common. Studies in other tumors have shown that the synchronous inhibition of BRAF and human murine double minute 2 homologue (MDM2 or HDM2) protein is more effective than BRAF monotherapy, particularly in the presence of wild type p53 (WTp53). To investigate the MDM2 protein expression and gene amplification in ameloblastoma, in association with BRAF^V600E^ and p53 expression. Forty-four cases of ameloblastoma fixed in 10% buffered formalin and embedded in paraffin were examined for MDM2 overexpression and BRAF^V600E^ and p53 expression by immunohistochemistry, and for *MDM2* ploidy with fluorescence in situ hybridization. Sixteen of forty-four (36.36%) cases of ameloblastoma showed MDM2 overexpression. Seven of sixteen MDM2-positive ameloblastomas (43.75%) were BRAF^V600E^ positive and fifteen of sixteen MDM2-positive ameloblastomas (93.75%) were p53 negative. All MDM2 overexpressing tumors did not show copy number alterations for MDM2. Overexpression of MDM2 in ameloblastomas is not associated with MDM2 amplification, but most probably with MAPK activation and WTp53 expression. Further verification of those findings could form the basis for the use of MDM2 expression as a marker of MAPK activation in ameloblastomas and the trial of dual BRAF/MDM2 inhibition in the management of MDM2-overexpressing/BRAFV600E-positive/WTp53 ameloblastomas.

## 1. Introduction

Ameloblastoma is a rare benign epithelial odontogenic neoplasm, i.e., a tumor originating from the tooth-forming epithelium. It is one of the most common odontogenic tumors and the commonest odontogenic neoplasm [1,2,3]. It is usually diagnosed in patients in the third to fifth decade of life, shows a slight predilection for males, and is preferentially localized in the mandible, with the angle of the mandible and the molar region being the most commonly affected regions [3,4]. Based on clinical, radiographic, and pathologic features, three types of ameloblastoma are described: the conventional solid/multicystic and the unicystic ameloblastoma are intraosseous tumors, whereas the peripheral ameloblastoma is an extraosseous tumor that develops in the gingiva. The conventional solid/multicystic ameloblastoma and the peripheral ameloblastoma are the most prevalent and the rarest types, respectively [3,4]. Clinically, conventional solid/multicystic ameloblastoma manifests among other symptoms and signs with swelling of the involved jaw region, mobility and displacement of teeth with consequent malocclusion, facial asymmetry, neurological signs, and bone fracture. Radiographically, it presents as a multilocular or unilocular radiolucency that may cause extensive bone destruction [3]. The diagnostic pathological feature of ameloblastoma is its resemblance to odontogenic epithelium, as it is composed centrally by cells resembling the stellate reticulum epithelium and peripherally by cylindrical, palisading cells with reverse nuclear polarity, resembling the ameloblasts. Pathological subtypes include follicular, plexiform, desmoplastic, basal cell, granular cell, and acanthomatous patterns, whereas the unilocular type is categorized into luminal, intraluminal, and mural subtypes, based on the localization of the tumor [5]. 

Although ameloblastoma is a benign, slow-growing tumor, it has an aggressive local behavior and high recurrence rate after conservative surgical techniques, such as enucleation alone or in combination with curettage, cryotherapy, cauterization, Carnoy solution, etc. [4,6,7]. On the other hand, radical surgical management techniques, such as segmental resection with wide bone margins or marginal resection, are associated with a lower recurrence rate, but also with severe complications and considerable decline in the patients’ quality of life [4,7,8]. Recurrences usually appear 2–5 years after treatment but may develop even 20 years later [4]. Furthermore, ameloblastomas extending into vital anatomical compartments, such as the base of the skull, orbit, and parapharyngeal space, pose additional difficulties in treatment, may be non-amenable to radical resection, and may even be life-threatening [9]. Therefore, there is a need for conservative treatment options, which are alternatives to or complementary to surgery. 

Ameloblastomas commonly show the activating mutation V600E in the *v-Raf* murine sarcoma viral oncogene homologue B (*BRAF^V600E^*) located on chromosome 7q34; BRAF is a key member of the mitogen-activated protein kinase (MAPK) signaling pathway, which plays a crucial role in cell growth and proliferation [10,11,12,13,14,15,16,17]. This finding prompted the clinical trial of BRAF inhibitors (BRAFi) as a targeted therapy in this tumor. BRAFi vemurafenib and dabrafenib have shown promising results in the management of *BRAF^V600E^*-positive ameloblastomas [18,19,20,21,22,23] or as neoadjuvant therapy [24,25,26]. In the largest series reported to date, 19 patients with *BRAF^V600E^*-positive ameloblastomas treated with dabrafenib ± trametinib achieved complete radiological response; one showed complete clinical response, and in ten of them residual tumor enucleation was possible with near complete or partial response [26]. However, the genetic heterogeneity of ameloblastoma, as well as variable responses and development of tolerance in targeted therapies [23], may limit the clinical success of such strategies.

Ameloblastomas commonly express the human murine double minute 2 homologue (MDM2 or HDM2) protein [27,28,29,30,31,32,33]. *MDM2* is located on chromosome 12q14.3–q15 and encodes the MDM2 E3 ubiquitin ligase [34,35]. MDM2 regulates cell growth and differentiation by binding ubiquitin to other proteins, such as the oncosuppressor proteins p53, pRB, and p14ARF [36,37,38,39,40], and FOXO3a transcription factor [41,42]. Its main function is to negatively regulate the wild-type p53 (WTp53) transcriptional activity [34,36,37,38,39,40,43,44] through its overexpression [45]. This results in p53-dependent arrest of cell proliferation and apoptosis [46], and it increases genomic instability [47]. MDM2 overexpression is commonly associated with *MDM2* gene amplification in several solid tumors, such as liposarcomas, osteosarcomas, breast and esophageal carcinomas, brain tumors, and neuroblastomas [40,46,47,48,49], while in cutaneous melanomas and hematological malignancies MDM2 overexpression is seen in the absence of *MDM2* amplification [49,50,51,52]. Blocking of *MDM2* expression, downregulation of MDM2 activity, or interference in the MDM2-p53 complex may reconstitute p53 function in cell cycle progression control and apoptosis promotion [49,52]. Therefore, MDM2 inhibitors have been tested in preclinical studies and some of them are under clinical investigation for solid tumors and hematological malignancies [49]. In some studies, synchronous inhibition of MDM2 and BRAF is more effective than BRAF monotherapy in *BRAF^V600E^*-positive melanomas [53,54,55].

In view of the complimentary roles of BRAFi and MDM2i in the treatment of various tumors, the objective of this study was to investigate MDM2 protein expression and gene amplification in ameloblastoma, in association with BRAF^V600E^ and p53 expression.

## 2. Results

Forty-four patients with ameloblastoma were included in this study; twenty-three were males and twenty-one females. The age range was 6–82 years and the mean age 42.6 ± 18.7 years. Thirty-five lesions were localized in the mandible and eight in the maxilla (mandible to maxilla ratio 4.3:1). Forty-one ameloblastomas were conventional solid/multicystic and three were unicystic. Solid ameloblastomas were follicular in twenty-seven cases, plexiform in six cases, basaloid in six cases, and acanthomatous in two cases. Unicystic ameloblastomas were of the mural subtype, all of them showing a follicular growth pattern. Squamous metaplasia, cystic degeneration, and granular cells were seen in five, ten, and one of the follicular ameloblastomas, respectively. 

### 2.1. Immunohistochemistry

**Figure 1:**
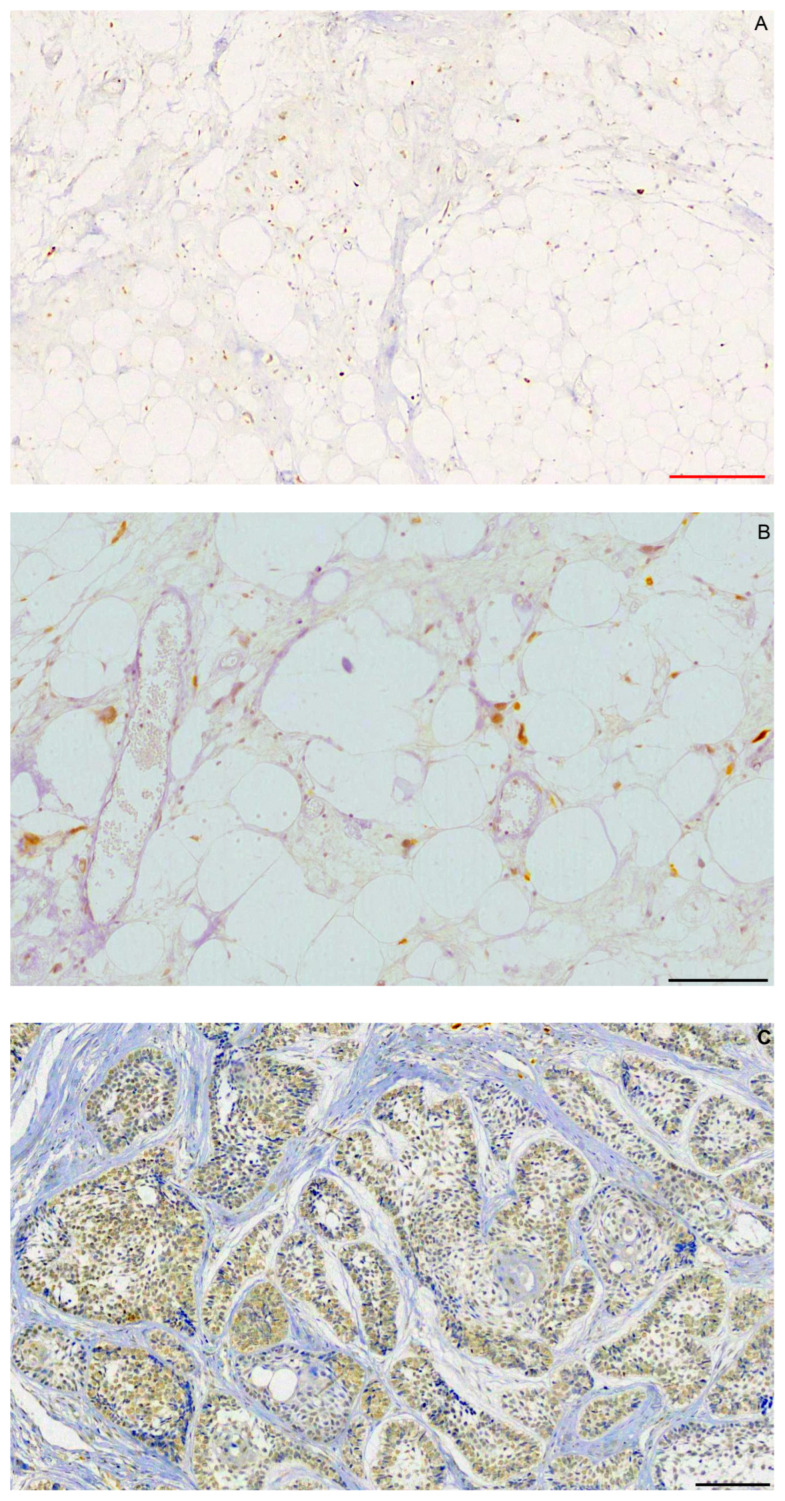
MDM2 strong (3+) nuclear immunostaining in (**A**,**B**) atypical lipomatous tumor and (**C**) follicular ameloblastoma. Scalebars: red = 50 μm, black = 100 μm.

Liposarcomas showed grade 3+ MDM2 immunostaining (Figure 1A,Β). In ameloblastomas, staining was 0 in 28 cases (63.63%), 2+ in 2 cases, and 3+ in 14 cases (Figure 1C). Therefore, all 16 (36.36%) MDM2-positive cases showed overexpression. No difference was observed in immunostaining between peripheral, ameloblast-like, and central, stellate reticulum-like cells. Squamous or granular cells did not react for MDM2. There was no statistically significant association (*p* < 0.05) between MDM2 expression and age or gender of the patients. 

**Figure 2:**
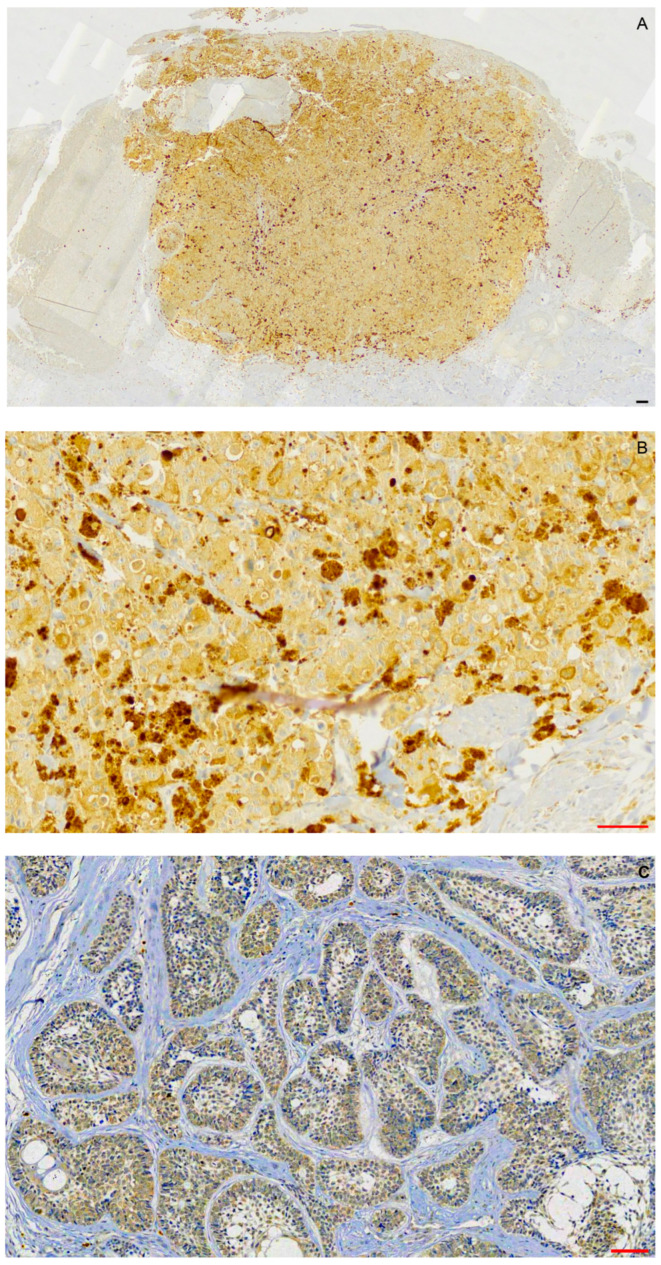
BRAF^V600E^ cytoplasmic immunostaining in (**A**,**B**) cutaneous melanoma and (**C**) follicular ameloblastoma. Scalebars: red = 50 μm, black = 100 μm.

Melanoma showed cytoplasmic, homogenous, and intense BRAF^V600E^ immunostaining in most tumor cells (Figure 2A,B). Seven of sixteen MDM2-positive ameloblastomas (43.75%) were BRAF^V600E^ positive (Figure 2C), two showed weak/ambiguous staining and were considered as negative, and seven were BRAF^V600E^ negative. All seven positive cases were from the mandible, representing seven of ten mandibular ameloblastomas and five of seven negative cases from the maxilla, and representing five of six maxillary ameloblastomas. Almost all (14/16) were of the follicular subtype. 

**Figure 3:**
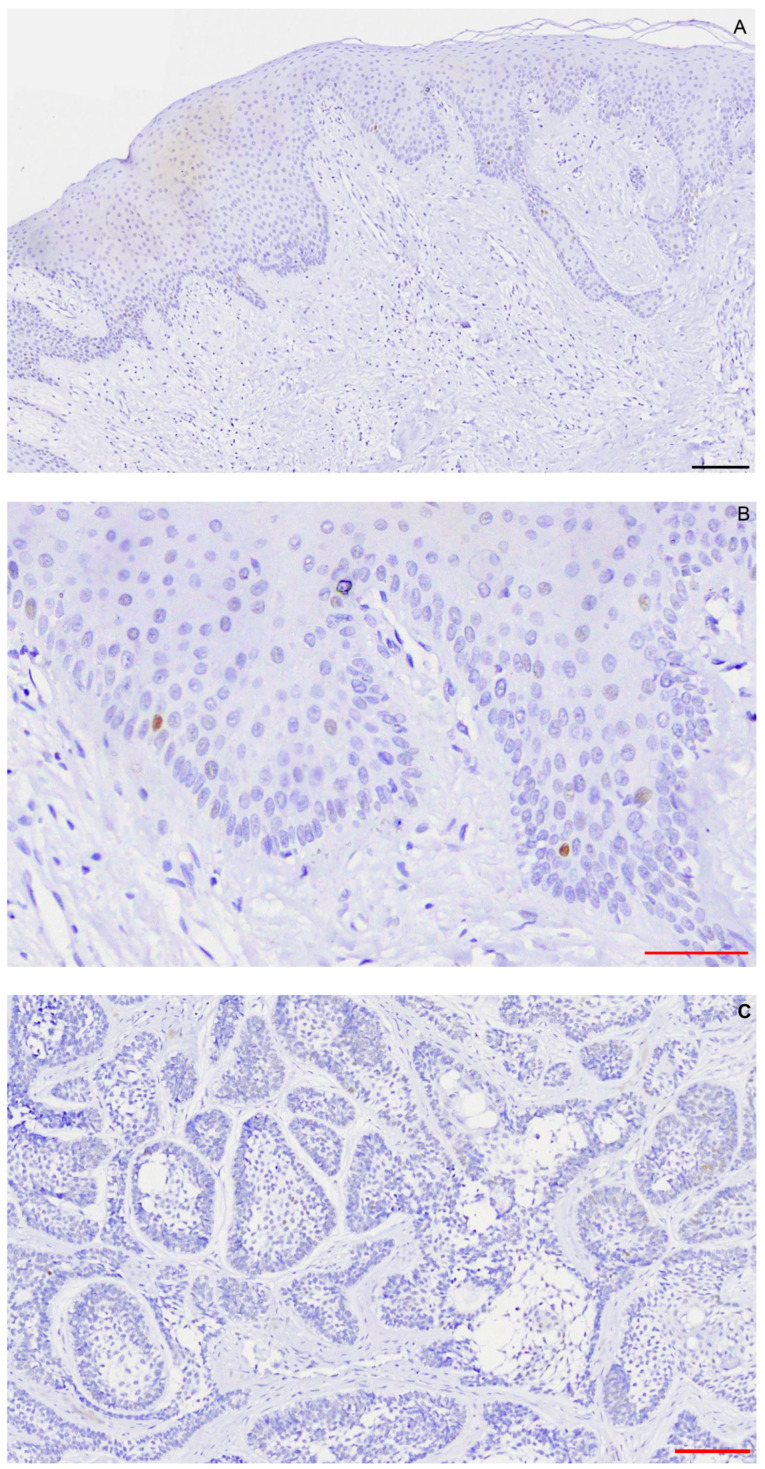
Scattered p53 nuclear immunostaining in gingival epithelium (**A**,**B**) and (**C**) in follicular ameloblastoma. Scalebars: red = 50 μm, black = 100 μm.

Specimens of normal oral mucosa showed nuclear, homogenous, and intense p53 immunostaining in rare cells of the basal cell layer (Figure 3A,B). Fifteen of sixteen MDM2-positive ameloblastomas (93.75%) were p53 negative and one was p53 positive (1+) (Figure 3C). Seven of sixteen ameloblastomas (43.75%) presented an MDM2-positive/BRAF^V600E^-positive/WTp53 phenotype. Figure 1C, Figure 2C and Figure 3C are from the same follicular ameloblastoma.

Table 1 shows the main clinical, histopathological, and immunohistochemical features of the 16 MDM2-positive ameloblastomas.

### 2.2. FISH

**Figure 4:**
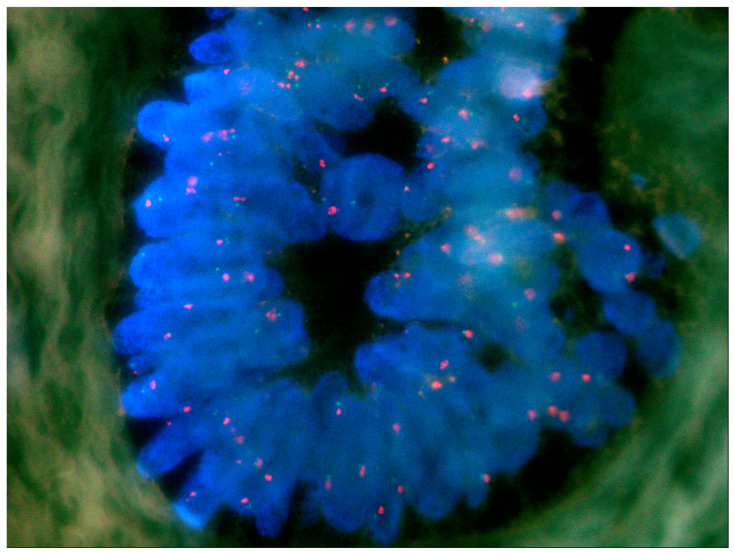
Fluorescence in situ hybridization (FISH) for MDM2 in a follicular ameloblastoma (ZytoLight-FISH tissue implementation kit). The orange signals represent the probe and the green the control probe. The presence of two orange and two green hybridized signals is representative of euploidy.

FISH was performed in the 16 MDM2-positive ameloblastomas. No copy number alterations for *MDM2* were identified in all tumors examined (Figure 4). 

## 3. Discussion

Herein, we aimed to investigate MDM2 protein expression and gene amplification in ameloblastoma, in association with BRAF^V600E^ and p53 expression. The main finding of the present study is that ameloblastomas overexpressing MDM2 did not show *MDM2* amplification, as accessed by FISH, whereas some of them expressed BRAF^V600E^ in the presence of WTp53, as was shown by immunohistochemistry. 

The immunohistochemical expression of MDM2 has been studied in ameloblastomas with inconsistent results. Carvalhais et al. [27] noticed weak nuclear reaction in eight of thirteen (61.53%) ameloblastomas. Sandra et al. [28] detected MDM2 in 33 of 34 (88%) and 32 of 34 (86%) ameloblastomas by immunohistochemistry and western blotting, respectively. In a study conducted by Kumamoto et al. [29], all 46 ameloblastomas expressed MDM2. Sharifi-Sistani et al. [30] found MDM2 expression in 31 of 39 (79.48%) ameloblastomas, Krishna et al. [31] in 33 of 36 (91.6%) ameloblastomas, and Singh et al. [32] in 18 of 20 (90%) of conventional solid/multicystic ameloblastomas and 12 of 20 (60%) unicystic ameloblastomas. Finally, Udeabor et al. [33] reported MDM2 expression in three of twenty-eight (10.7%) ameloblastomas. The varying results on MDM2 immunoexpression among different studies may be attributed to technical parameters [47], but overall MDM2 expression was recorded in 181 of 226 ameloblastomas tested. In this study, MDM2 positivity, defined as nuclear immunostaining [56,57] that is homogenous and clear [58,59], was seen in 16 of 44 cases (36.36%). 

In accordance with previous reports, MDM2-positive cells were seen in all cell layers [27], squamous or granular cells were MDM2 negative [31], and no difference was observed between follicular and plexiform ameloblastomas [27]. Other studies have reported more intense MDM2 expression in the peripheral cells of ameloblastoma [28,29,32] and variation among the histopathological types [29,30,31,32], findings that were not confirmed in the present material.

MDM2 overexpression is considered suggestive of *MDM2* amplification in various malignant tumors, such as bladder carcinoma, melanoma, and liposarcoma [51,56,57], but it has not been evaluated in ameloblastomas. In this study, all MDM2-positive cases showed MDM2 overexpression, whereas in previous studies none [28,33], 38.46% [27], or 61.54% [30] of the tumors examined showed immunohistochemical expression consistent with overexpression. However, none of the 16 MDM2-overexpressing tumors showed *MDM2* amplification by FISH. Those findings are in line with previous studies [10,12,13,60,61] that by employing various molecular techniques, e.g., microarrays, RNA-sequencing, Sanger sequencing, and polymerase chain reaction (PCR), highlighted the differential expression and/or mutations of other molecular markers that do not predict *MDM2* mutations in ameloblastoma.

MDM2 overexpression in the absence of *MDM2* amplification has been reported in malignant melanoma [50,51,52], Burkitt lymphoma [62], carcinoma of the breast [63,64], carcinoma of the bladder [65], and soft tissue sarcomas [66]. It may be attributed to alternative splicing or increased transcription of *MDM2*, with the latter being the leading cause [34,67,68]. Increased *MDM2* transcription may be stimulated by the MAPK and transforming growth factor-β (TGF-β) pathways, as *MDM2* is a transcriptional target of both pathways [34,69]. 

MDM2 transcription may be activated by binding on its P2 promoter, located in the first intron of the molecule, by Activator protein 1 (AP-1) and E26 transformation-specific or E-twenty-six (ETS) transcription factors that are downstream molecules of the BRAF pathway [34]. The MAPK pathway may be constitutively activated by *BRAF^V600E^* mutation [51] that is frequently detected in ameloblastomas [10,11,12,13,14,15,17]. Seven of sixteen MDM2-positive cases in the present study were shown by immunohistochemistry to be positive for BRAF^V600E^ (43.75%), compared with the reported 46% to 82% positivity for this marker in other studies [10,11,12,13,14,15,17], and most of them were in the mandible [10,17]. Although the gold standards for detecting *BRAF^V600E^* mutation are PCR and DNA sequencing, immunohistochemistry with VE1 antibody, as applied in the present study, shows high concordance with molecular techniques [12,70]. TGF-β upregulates MDM2 expression via the interaction of Smad2 and Smad3 with the P2 promoter located in the first intron of *MDM2* [34]. In ameloblastomas, low expression of TGF-β1 and functional pSmad2/3 and Smad4 proteins do not indicate a critical role for the TGF-β pathway [71]. Those findings support the suggestion that MAPK activation through *BRAF^V600E^* mutation may be the main cause of MDM2 overexpression in BRAF^V600E^-positive tumors. As for BRAF^V600E^-negative cases that overexpress MDM2, it should be noticed that MAPK-activating mutations other than *BRAF^V600E^* have been identified in ameloblastomas [16], and in some ameloblastomas the TGF-β pathway may be activated [71]. Further evaluation of MDM2 overexpression/MAPK activation association could show its possible utility as a marker of MAPK activation.

In ameloblastoma, *TP53* mutations are rare [29,72,73]; p53 is mostly normal WTp53 [28], and there is heavy suppression of p53 [60]. In accordance with those studies, the present one showed that p53 was not expressed in most ameloblastomas with MDM2 overexpression, indicating p53 suppression. 

In cutaneous melanomas that overexpress MDM2 without *MDM2* amplification and which have normal p53, inhibition of MDM2 may reconstitute WTp53 action in tumor cells and suppress tumor growth [52]. Furthermore, MDM2i may act as a complement to BRAFi in malignant neoplasms that overexpress MDM2, are BRAF^V600E^ positive, and express WTp53. Dual BRAF/MDM2 inhibition suppressed the viability of WTp53 melanoma cells in vitro and WTp53 melanoma growth in vivo [54], and in cell cultures of cutaneous melanoma [74,75,76] and colon carcinoma [77] this led to restoration of p53 function, possibly promoting apoptosis and suppressing tumor growth. In mice xenografted with RKO colon carcinoma cell inhibitors of BRAF and MDM4, a nuclear protein structurally homologous to MDM2 that interacts with both p53 and MDM2, this treatment managed to shrink the tumor by 80%, when the response to each drug tested separately was 23% and 24%, respectively [77]. Furthermore, dual inhibition helped overrun tolerance to BRAFi, an adverse effect attributed to reactivation of the MAPK pathway [22,53,54]. In *BRAF^V600E^*-positive ameloblastomas which were unresectable due to multiple recurrences and lung metastases, monotherapy with BRAFi dabrafenib [19,21] or vemurafenib [20], neoadjuvant treatment with dabrafenib [24,25,26], or dual BRAF/MEK inhibition with dabrafenib and trametinib [18,22,23,26] showed good response without severe toxicity. The addition of MDM2i such as nutlins, which disrupt the MDM2-p53 interaction by competing with p53 for binding to the MDM2 protein, could augment the therapeutic outcome and possibly overcome tolerance to BRAFi [23]. 

A limitation of the present study is that BRAF^V600E^ and p53 expression were not examined in MDM2-negative ameloblastomas of our sample, as the investigation was focused on MDM2-overexpressing ameloblastomas. Therefore, the association of BRAF^V600E^ and p53 expression with MDM2 status cannot be concluded, although for the latter it is expected, based on the available literature, that the tumors would be p53 negative.

## 4. Materials and Methods

The cohort consisted of 44 biopsies of ameloblastoma fixed in 10% buffered formalin and embedded in paraffin (FFPE). Histopathologic diagnosis in each case was confirmed by microscopic examination of 5 μm thick FFPE tissue sections stained with hematoxylin and eosin by all researchers according to the World Health Organization diagnostic criteria for ameloblastoma [5]. Relevant clinical information was retrieved from the pathology request forms, tabulated, and anonymized. The study was approved by the Ethics Committee of the Dental School, National and Kapodistrian University of Athens (#302), and the Institutional Review Board of the University of Minnesota (IRB #1604E86681), and was conducted in accordance with the principles of the Declaration of Helsinki.

### 4.1. Immunohistochemistry

Immunohistochemistry was performed on 5 μm thick FFPE tissue sections utilizing the OptiView DAB IHC Detection Kit (Ventana Medical Systems Inc., Tucson, AZ, USA). Staining was performed in the fully automated VENTANA BenchMark ULTRA Slide Staining System (Ventana Medical Systems Inc., Tucson, AZ, USA) with mouse anti-human monoclonal antibodies against MDM2 (1:50, Clone IF2, Invitrogen Corporation, Camarillo, CA, USA) that recognize an epitope between amino acids 26–169 of human MDM2; recombinant mouse monoclonal antibody to BRAF-mutated V600E (1:50, clone VE1, (1:50, clone VE1, Abcam, Amsterdam, Netherlands); and mouse anti-human monoclonal antibodies against p53 (1:300, clone DO7, Biogenex, Fremond, CA, USA). The staining reaction was visualized with 0.2% 3,3′-diaminobezidine solution solubilized with OptiView DAB (Ventana Medical Systems Inc., Tucson, AZ, USA). For antigen retrieval, sections were treated in 97 °C for 25 min with ULTRA Cell Conditioning Solution (ULTRA CC1, Ventana, Roche Diagnostic GmbH, Manheim, Germany) for all antibodies.

MDM2, BRAF^V600E^, and p53 were evaluated in one representative 5 μm FFPE tissue section from each tumor, digitized with a semi-automated system with Intel Pentium V, Digital Camera Sony 1600 × 1200, and Microscope Olympus CX-31 hardware features, and the software Windows XP/Windows XP/NIS-Elements Software AR v3.0, Nikon Corp, Tokyo, Japan. The 5 μm FFPE tissue section of each sample was scanned, resulting in a digital image of 20x original magnification. Scoring for MDM2 was performed semi-quantitatively based on the pattern of nuclear staining with the following scale: 0 < 5%; 1+ = 5–20%; 2+ = 21–50%; and 3+ > 50% [57]; overexpression was defined as ≥2+ [51,56,57]. Scoring for BRAF^V600E^ was based on the intensity of cytoplasmic staining, with the following scale: 0, negative; 1+, weak; 2+, moderate; and 3+, strong [78], and the threshold for p53 positivity was 5%. 

Positive controls were sections from two cases of atypical lipomatous tumor of the thigh for MDM2, two cases of oral mucosa for p53, and one case of *BRAF^V600E^*-positive cutaneous melanoma for VE1. For negative controls, the primary antibodies were substituted with Negative Control-monoclonal (Ventana, Medical Systems Inc., Tucson, AZ, USA).

### 4.2. FISH

FISH was performed on 5 μm thick FFPE tissue sections with the commercially available Zyto*Light*-FISH tissue implementation kit and Zyto*Light*-FISH SPEC MDM2/CEN12 Dual Color Probe (ZytoVision® GmbH, Bremerhaven, Germany). This is a direct labeling technique optimized for use with FFPE tissue sections, with ready-to-use fluorescence-labeled polynucleotide probes: a green one targeting the chromosomal region of the human MDM2, and an orange one targeting DNA sequences of centromeric alpha-satellites of chromosome 12 (CEN12). Alpha-satellite sequences of CEN12 served as an internal control and as a measure for DNA integrity. Sections were examined with an oil-immersion ×100 lens and proper fluorescence filters (green-labeled polynucleotides: excitation at 503 nm and emission at 528 nm, orange-labeled polynucleotides: excitation at 547 nm and emission at 572 nm). Amplification of the *MDM2* gene locus was defined as an MDM2/CEN12 signal ratio ≥2 in >10% of the total number of cells or as clustering of multiple copies of green signals [79,80]. In ameloblastomas, CEN12 was expected to be euploid, as chromosomal copy number variations for this tumor are rare and do not include chromosome 12 [73,81,82,83,84]. Therefore, no external controls were necessary. Fifty interphase nuclei from different areas of the FISH slides were evaluated in each case.

### 4.3. Statistical Analysis

Statistical analysis was performed with the SPSS, v25.0 Software for Windows (SPSS Inc., Chicago, IL, USA). Associations between the MDM2 immunohistochemistry results and patients’ demographic characteristics were investigated with the Chi Square Test and, when expected frequency was <5, with the Fisher Exact Test. The level of statistical significance was set at *p*-value (*p*) < 0.05.

## 5. Conclusions

In conclusion, overexpression of MDM2 in ameloblastomas is not associated with *MDM2* amplification, but most probably with MAPK activation and WTp53 expression. Further verification of those findings could form the basis for the use of MDM2 expression as a marker of MAPK activation in ameloblastomas and the trial of dual BRAF/MDM2 inhibition in the management of MDM2-overexpressing/BRAF^V600E^-positive/WTp53 ameloblastomas.

## Figures and Tables

**Table 1:** Main clinical, histopathological, and immunohistochemical features of 16 MDM2-positive ameloblastomas.

Gender	Age	Jaw	Histological Subtype	MDM2	BRAF^V600E^	p53
M	52	maxilla	reticular	3	1	-
M	33	mandible	follicular	3	1	-
F	37	mandible	follicular	3	1	+
M	45	maxilla	reticular	3	1	-
F	55	maxilla	follicular	3	0	-
M	61	maxilla	follicular	3	1	-
M	46	mandible	follicular	2	0	-
M	49	mandible	follicular	2	0	-
M	24	mandible	follicular	3	0	-
F	77	mandible	follicular	3	1	-
M	67	mandible	follicular	3	1	-
F	31	mandible	follicular	3	0	-
F	55	mandible	follicular	3	0	-
M	43	maxilla	follicular	3	0	-
F	41	maxilla	follicular	2	0	-
M	38	mandible	follicular	3	0	-

## Data Availability

The original contributions of the study are included in the article. Further inquiries can be directed to the corresponding author.
